# MHD mixed convection and heatlines approach of nanofluids in rectangular wavy enclosures with multiple solid fins

**DOI:** 10.1038/s41598-023-36297-9

**Published:** 2023-06-14

**Authors:** Fatin M. Azizul, Ammar I. Alsabery, Ishak Hashim, Rozaini Roslan, Habibis Saleh

**Affiliations:** 1 Department Mathematical Sciences, Faculty of Science & Technology, Universiti Kebangsaan Malaysia, UKM, 43600 Bangi, Selangor Malaysia; 2 Refrigeration & Air-conditioning Technical Engineering Department, College of Technical Engineering, The Islamic University, Najaf, Iraq; 3 Nonlinear Dynamics Research Center (NDRC), Ajman University, Ajman, United Arab Emirates; 4 Department of Mathematics & Statistics, Faculty of Applied Sciences & Technology, Universiti Tun Hussein Onn Malaysia, 84600 Pagoh, Muar Malaysia; 5 Mathematics Education Department, Universitas Islam Negeri Sultan Syarif Kasim Riau, Pekanbaru, 28293 Indonesia

**Keywords:** Mechanical engineering, Computational nanotechnology, Applied mathematics, Software

## Abstract

Two dimensional wavy walls rectangular cavity with inclined magnetohydrodynamic has been examined in mixed convection configurations. Triple fins arranged in the upwards ladder were filled within alumina nanoliquid in the cavity. Vertical sinusoidal walls were heated, and the other side was kept cold while both horizontal walls were kept adiabatic. All walls were motionless except the top cavity that was driven to the right. The diversified range of control parameter in Richardson number, Hartmann number, number of undulations, length of the cavity has been performed in this study. The analysis was simulated using finite element method by employing the governing equation formula, and the results were delineated in the form of streamlines, isotherms, heatlines, and comparisons on several relationships between the local velocity in the *y*-axis line of 0.6, local and average Nusselt number along the heated surface and dimensionless average temperature. The findings revealed that high concentration nanofluids boost the rate of heat transfer without the need to apply any magnetic field. Results found that the best heat mechanisms are natural convection with significant-high Richardson number as well as constructing two waves on the vertical walls in the cavity.

## Introduction

The buoyancy force’s created from different temperature regions and lid-driven force from the wall’s movement taking place in the same system can be acknowledged as mixed convection. Mixed convection is interrelated with Richardson number, *Ri*, which originates from Grashof number and Reynolds number. Many studies agreed that altering *Ri* can modify the types of convection in investigated and related factors related to various cases such as home ventilation, fire prevention, solar collector, drying technologies, and chemical processing, as indicated by^1^. The rectangular shape is chosen in this present study due to limited discussion on such cases in the literature and it is based on original idea from^2^. They constructed a rectangular model of open-loop air supplied heating system by modifying the jet injection mode. Then,^3^ and^4^ conducted mixed convection in the rectangular enclosure by inserting a centrally located continuous moving horizontal plate and filled the cavity with fluid-saturated porous medium, respectively. To make this problem more interesting,^5^ embedded a heated elliptical block in the rectangular cavity, and the result showed that the block provided better heat transfer compared to a circular cylinder. Next, an analysis of unsteady mixed convection consisting two rectangular fins was simulated by^6^. They stated that the size of the fins influenced the performance of heat energy in the system. Additionally,^7^ also concentrated on natural convection in rectangular enclosure that featured with several heat sources. The outcomes showed the magnetic field angle rises as the number of heat sources in experiment boost the heat transfer efficiency.

Although the discussion on wavy wall is a typical issue in natural and forced convection^8–15^, the case on double-sided undulation on mixed convection still lacks attention. In 2013,^16^ was the first to design two wavy surfaces in a filled vertical rectangular cavity for the purpose of examining the type of nanofluids, nanoparticle fraction and other parameters. They declared that copper nanoparticle with high volume fraction achieved high average Nusselt number. After some time,^17^ used a fractional partial differential equation to solve mixed convection problem involving hybrid nanofluid in a porous inclined cavity that has two surfaces with inlet and outlet in the lower part. The finding presented that deeper wavy amplitude with high Rayleigh number improves the rate of heat transfer.

The existence of advanced technology motivates many researchers to build a better heat transfer configuration, for example, in building design, house construction, and so forth. Thus, they proposed a variety of shapes and complex geometries by adjusting the model and inserting different kinds of objects inside the system. One of them is^18^ who applied triangular fins to a stationary wall in a square cavity mixed convection problem. They studied the effect of the fins’ location and figured out that the fins weaken the streamline and reduce local Nusselt number. Then, a three-dimensional mixed convection was conducted by^19^, which placed longitudinal triangular fins at the middle walls of the bottom, left and right sections. The outcome presented that to obtain the maximum possible heat transfer and entropy generation, any type of case with top wall lid to the right can be used. Next,^20^ investigated on mixed convection of the two vertical walls cavity that are occupied with alumina-nanofluid and with a rotating cylinder positioned at the middle. Their studies proved that the level of nanofluid concentration and length of heater at the bottom cavity intensify heat performance. In addition, an elastic vertical fin attached at the upper wall was performed by^21^. They stated that the installation of fins enhanced the average Nusselt number as Richardson number increases. Saleh et al. ^22^ applied a heated circular cylinder on mixed convection in a square cavity with flexible fins attached at the upper wall and outer surfaces were kept cold. Their study is considered unsteady and solved by using Arbitrary Langrangian-Eulerian resulting that the fins motion, cylinder size and elasticity affect the fluid flow and convective strength.

Furthermore, magnetohydrodynamics (MHD) is carried out in electrically conducting fluids to produce a magnetic field, manipulated by the Hartmann number. A study by^23^ listed that MHD is utilised in heat exchangers, chemical reactors, electrical devices, and stratified atmospheric boundary layers. MHD is unique in the sense that it can effectively be inserted in the system horizontally, vertically, and in an inclined direction. Bakar et al.^24^ applied the uniform magnetic horizontally and discovered that the Hartmann number’s appearance reduces convective heat flow and rate of heat transfer. This finding were strongly supported by^25^ and^26^. Moreover, the vertical direction magnetic field with mixed convection of different heated vertical walls in the square cavity was investigated by^27^. They also agreed that the existing magnetic field weakens the fluid’s velocity and conduction dominates the model. Many research conducted studies on the problem of inclined magnetic field with nanofluids filled in close enclosure of mixed convection such as^19–31^. All of them declared that the magnetic field’s inclination angle induced provided superior parameters by controlling fluid velocity, temperature behaviour, heat performance, and others. A nanometer sized particle was used due to its high stability and thermal conductivity. Findings from^1–38^ acknowledged that nanofluid is better in augmenting heat performance than conventional base fluid. The earliest idea to increase the volume fraction of solid particle was made by^39^. A review by^40^ then affirmed that utilisation of MHD in nanofluids could be found in manufacturing, electrical, electronics, automotive, biomedical, and so on.

Finally, heat transport can be visualised between the collaboration of fluid path velocity and temperature fluid flow in the system, as initiated by^41^. The special name used among the scholars is heatline visualisation and is produced by the simulation of heat function. Apart from seeing the result of streamlines, isotherms, entropy generation, nanoparticle distribution, heatline can also be essential in observing heat energy movement^42^. Finite element method is the preferred method used to simulate this matter. For example,^43,44^ applied this method for mixed convection by varying the direction of the lid-driven wall in basic square cavities and porous square cavities, respectively. Narayana^45^ used heatline technique to investigate mixed convection in a half heated square cavity with the top surface being isothermal and moved to the right. Their study used normalised variable diagram (NVD) in getting better boundedness and accurate simulation. A problem involving heatline on mixed convection with porous fins was elaborated by^46^. More fins with low Darcy number and small Richardson number can improve the rate of heat transfer by placing the fins nearer to the bottom sidewall. Next,^47^ also conducted mixed convection in a heated wavy cavity with isothermal inner block, and they revealed that inner block with a size of 0.3 cm and four sinusoidal surfaces at the bottom contributed to optimum heat performance.

Based on the literature analysis, investigation on mixed convection using two wavy surfaces with fins inside the cavity is still lacking. Hence, this study proposed a mixed convection model in a rectangular lid-driven wavy cavity with the effect of inclined magnetic field and alumina nanoparticle. Not only that, uniqueness and complexities verifications were done by compiling triple horizontal rectangular fins in step upwards order without attaching to any walls. Other factors are also included such as Richardson and Hartmann number, volume fraction nanoparticle, and cavity length. This work also elaborated the results clearly using illustrations and graphs on fluid velocity, temperature behaviour, heat transportation, local velocity, local and average Nusselt number, and dimensionless average temperature.

## Mathematical formulation

**Figure 1:**
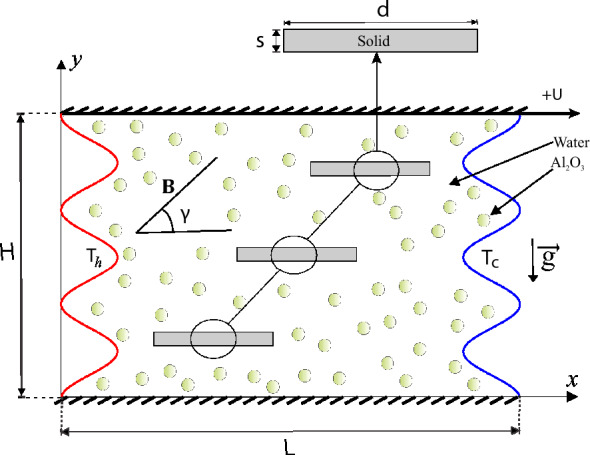
The study’s problem model geometry accompanied by the coordinate system.

An illustration of the present work is designed in a rectangular wavy cavity shown in Fig. 1. The Al$_2$O$_3$-nanoliquids and uniform magnetic field with magnitude $\text {B}$ and angle $\gamma$ are implemented within the wavy cavity. A sinusoidal shape is built at vertical walls and isothermally heated with left and right is $T_h$ and $T_c$, respectively and single constant velocity with $+U$ at the upper surface while the other walls are maintained stationary. Besides, three solid fins with a height *s* and width *d* are placed in the staircase order in the middle cavity without sticking to the wall. The model is considered to be two-dimensional, steady-state, laminar, and incompressible viscous flow. Standard governing Navier-Stokes equations (continuity, momentum and energy equation) of the Newtonian water-liquid are symbolised as follows:

1 $$\begin{aligned} {\frac{{\partial u}}{{\partial x}}} + {\frac{{\partial v}}{{\partial y}}}{} & {} = 0 \end{aligned}$$

2 $$\begin{aligned} u\frac{{\partial u}}{{\partial x}} + v\frac{{\partial u}}{{\partial y}}{} & {} = - \frac{1}{{{\rho _{nf}}}}\frac{{\partial p}}{{\partial x}} + {\nu _{nf}}\,\left( {\frac{{{\partial ^2}u}}{{\partial {x^2}}} + \frac{{{\partial ^2}u}}{{\partial {y^2}}}} \right) \nonumber \\{} & {} \quad + \frac{\sigma _{nf}\text {B}^2}{\rho _{nf}} \left( v\sin \gamma \cos \gamma -u\sin ^2\gamma \right) , \end{aligned}$$

3 $$\begin{aligned} u\frac{{\partial v}}{{\partial x}} + v\frac{{\partial v}}{{\partial y}}{} & {} = - \frac{1}{{{\rho _{nf}}}}\frac{{\partial p}}{{\partial y}} + {\nu _{nf}}\,\left( {\frac{{{\partial ^2}v}}{{\partial {x^2}}} + \frac{{{\partial ^2}v}}{{\partial {y^2}}}} \right) + \beta _{nf} \, g(T - {T_c}) \nonumber \\{} & {} \quad + \frac{\sigma _{nf}\text {B}^2}{\rho _{nf}} \left( u\sin \gamma \cos \gamma -v\cos ^2\gamma \right) , \end{aligned}$$

4 $$\begin{aligned} u\frac{{\partial T}}{{\partial x}} + v\frac{{\partial T}}{{\partial y}}{} & {} = {\alpha _{nf}}\left( {\frac{{{\partial ^2}T}}{{\partial {x^2}}} + \frac{{{\partial ^2}T}}{{\partial {y^2}}}} \right) \end{aligned}$$

The heat equation of the solid fins remains as:

5 $$\begin{aligned} \frac{{{\partial ^2}T_s}}{{\partial {x^2}}} + \frac{{{\partial ^2}T_s}}{{\partial {y^2}}} = 0, \end{aligned}$$

Here, *x* and *y* obtain the Cartesian coordinates that aligned in the horizontal and vertical directions sequentially, *g* holds the acceleration due to gravity, $\rho _{nf}$ denotes the density of the nanofluid and $\nu _{nf}$ is the kinematic viscosity of the nanofluid.

The thermophysical characteristics concerning the nanofluid can be indicated as^48,49^:

6 $$\begin{aligned} \alpha _{nf}{} & {} =\frac{k_{nf}}{(\rho C_p)_{nf}}, \end{aligned}$$

7 $$\begin{aligned} \rho _{nf}{} & {} =(1-\phi )\rho _{f}+\phi \rho _{p}, \end{aligned}$$

8 $$\begin{aligned} {(\rho \beta )_{nf}}{} & {} = (1 - \phi ){(\rho \beta )_{f}} + \phi {(\rho \beta )_{p}}, \end{aligned}$$

9 $$\begin{aligned} (\rho C_p)_{nf}{} & {} =(1-\phi )(\rho C_p)_{f}+\phi (\rho C_p)_{p}, \end{aligned}$$

10 $$\begin{aligned} \frac{\mu _{nf}}{\mu _f}{} & {} = \frac{1}{\left( 1-34.87\left( \frac{d_p}{d_f}\right) ^{-0.3}\phi ^{1.03}\right) }, \end{aligned}$$

11 $$\begin{aligned} \frac{k_{nf}}{k_{f}}{} & {} = 1 + 4.4 \text {Re}_B^{0.4} \text {Pr}^{0.66} \left( \frac{T}{T_{fr}}\right) ^{10} \left( \frac{k_p}{k_f}\right) ^{0.03} \phi ^{0.66}, \end{aligned}$$

12 $$\begin{aligned} \frac{\sigma _{nf}}{\sigma _{f}}{} & {} = 1+ \frac{3 \left( \frac{\sigma _{p}}{\sigma _{f}}-1\right) \phi }{\left( \frac{\sigma _{p}}{\sigma _{f}}+2\right) - \left( \frac{\sigma _{p}}{\sigma _{f}}-1\right) \phi }. \end{aligned}$$

Presently we propose the next adopted non-dimensional variables:

13 $$\begin{aligned} X{} & {} =\frac{x}{L},\quad Y=\frac{y}{L},\quad U = \frac{u}{U_{0}}, \,\, V = \frac{v}{U_{0}},\quad \theta =\frac{T - T_c}{T_h - T_c},\nonumber \\ D{} & {} =\frac{d}{L}, \quad H=\frac{h}{L}, \quad B=\frac{b}{L}, \quad \Pr = \frac{\nu _f}{\alpha _f}, \quad P=\frac{pL^2}{\rho _{f} \alpha _f^2}, \nonumber \\ Gr{} & {} =\frac{g\beta _{f}(T_{h}-T_{c})L^3}{{\nu _{f}}^2}, \quad Re=\frac{U_{0}L}{\nu _f}, \quad Ri=\frac{Gr}{Re^2}, \quad Ha=\text {B} L \sqrt{\frac{\sigma _f}{\mu _f}}, \end{aligned}$$

Using the above parameter yields the dimensionless governing equations as below:

14 $$\begin{aligned} \frac{{\partial U}}{{\partial X}} + \frac{{\partial V}}{{\partial Y}}{} & {} = 0 \end{aligned}$$

15 $$\begin{aligned} U\frac{{\partial U}}{{\partial X}} + V\frac{{\partial U}}{{\partial Y}}{} & {} = - \frac{{\partial P}}{{\partial X}} + \frac{\rho _{f}}{\rho _{nf}} \frac{\mu _{nf}}{\mu _{f}} \frac{1}{Re} \left( {\frac{{{\partial ^2}U}}{{\partial {x^2}}} + \frac{{{\partial ^2}U}}{{\partial {Y^2}}}} \right) \nonumber \\{} & {} \quad + \frac{\rho _{f}}{\rho _{nf}} \frac{\sigma _{nf}}{\sigma _{f}} \frac{Ha^2}{Re} \left( V\sin \gamma \cos \gamma -U\sin ^2\gamma \right) , \end{aligned}$$

16 $$\begin{aligned} U\frac{{\partial V}}{{\partial X}} + V\frac{{\partial V}}{{\partial Y}}{} & {} = - \frac{{\partial P}}{{\partial Y}} + \frac{\rho _{f}}{\rho _{nf}} \frac{\mu _{nf}}{\mu _{f}} \frac{1}{Re} \left( {\frac{{{\partial ^2}V}}{{\partial {X^2}}} + \frac{{{\partial ^2}V}}{{\partial {Y^2}}}} \right) + \frac{(\rho \beta )_{nf}}{\rho _{nf} \beta _{f}} {Ri}\, {\theta } \nonumber \\{} & {} \quad + \frac{\rho _{f}}{\rho _{nf}} \frac{\sigma _{nf}}{\sigma _{f}} \frac{Ha^2}{Re} \left( U\sin \gamma \cos \gamma -V\cos ^2\gamma \right) , \end{aligned}$$

17 $$\begin{aligned} U\frac{{\partial \theta }}{{\partial X}} + V\frac{{\partial \theta }}{{\partial Y}}{} & {} = \frac{\alpha _{nf}}{\alpha _{f}} \frac{1}{{\Pr }\, {Re}} \left( \frac{{{\partial ^2}\theta }}{{\partial {X^2}}} + \frac{{{\partial ^2}\theta }}{{\partial {Y^2}}}\right) , \end{aligned}$$

18 $$\begin{aligned} \frac{{{\partial ^2}\theta _s}}{{\partial {x^2}}} + \frac{{{\partial ^2}\theta _s}}{{\partial {y^2}}}{} & {} = 0, \end{aligned}$$

The dimensionless boundary conditions regarding Eqs. (14) and (18) are in the following forms:

19 $$\begin{aligned}{} & {} \text {On the top moving adiabatic wall:} \nonumber \\{} & {} U = 0, V = 1,\,\, \frac{\partial \theta }{\partial X}=0, \,\, 0\le X \le 1, \,\, Y=1 \end{aligned}$$

20 $$\begin{aligned}{} & {} \text {On the bottom adibatic wall:} \nonumber \\{} & {} U = V = 0,\,\, \frac{\partial \theta }{\partial X}=0,\,\, 0\le X \le 1,\,\, Y=0, \end{aligned}$$

21 $$\begin{aligned}{} & {} \text {On the left hot wavy wall:} \nonumber \\{} & {} U = V = 0,\,\, \theta =1,\,\, X= A(1-\cos (2N\pi Y)), \,\, 0\le Y \le 1 \end{aligned}$$

22 $$\begin{aligned}{} & {} \text {On the right cold wavy wall} \nonumber \\{} & {} U = V = 0,\,\, \theta =0,\,\, X= 1.5- A(1-\cos (2N\pi Y)), \,\, 0\le Y \le 1 \end{aligned}$$

The dimensionless pattern concerning the heatfunction (*H*) from the investigated problem can be achieved as^50^:

23 $$\begin{aligned} \frac{\partial H}{\partial Y}= U\theta - \frac{\alpha _{nf}}{\alpha _{f}} \frac{1}{{\Pr }\,{Re}} \frac{\partial \theta }{\partial X}, \quad -\frac{\partial H}{\partial X}= V\theta - \frac{\alpha _{nf}}{\alpha _{f}} \frac{1}{{\Pr }\,{Re}} \frac{\partial \theta }{\partial Y}, \end{aligned}$$

which generates a single equation

24 $$\begin{aligned} \frac{\partial ^2 H}{\partial X^2}+\frac{\partial ^2 H}{\partial Y^2}=\frac{\partial }{\partial Y} (U\theta ) - \frac{\partial }{\partial X} (V\theta ). \end{aligned}$$

Affected by the hot or cold isothermal walls, Neumann boundary condition of the heatfunction based on Eq. (23) plus the normal derivatives ($\text {n} \cdot \nabla H$) are identified as the following:

25 $$\begin{aligned} \text {n} \cdot \nabla H = 0 \quad \quad \text {(heated/cooled wall)}. \end{aligned}$$

Dirichlet boundary condition that treated Eq. (23) is described toward the top adiabatic surface which is clarified into $\frac{\partial H}{\partial Y}$. A reference rate of *H* exists to be zero through $X=0$, $Y=1$ plus hence $H=0$ is adequate for $Y=1$ $\forall X$. This indicates that the individual solution of Eq. (25) is completely directed to the non-homogeneous Dirichlet condition. The boundary conditions on the crossing point of the hot wavy and cold vertical surfaces are applied to achieve the solution of Eq. (23),

26 $$\begin{aligned}{} & {} H(0,0) = \frac{k_{nf}}{k_f} \frac{1}{{\Pr }\, {Re}} \overline{Nu}_l,\quad \text {(On the left surface)} \end{aligned}$$

27 $$\begin{aligned}{} & {} H(1,0) = - \frac{k_{nf}}{k_f} \frac{1}{{\Pr }\, {Re}} \overline{Nu}_r.\quad \text {(On the right surface)} \end{aligned}$$

The local Nusselt number estimated for the heated left wavy surface is obtained to estimate the heat transfer enhancement by:

28 $$\begin{aligned}{} & {} Nu_{nf} = \frac{h\, L}{k_{nf}} = - \frac{k_{nf}}{k_f}\frac{\partial \theta }{\partial n}L, \end{aligned}$$

29 $$\begin{aligned}{} & {} \frac{\partial \theta }{\partial n} = \frac{1}{L} \sqrt{\left( \frac{\partial \theta }{\partial X}\right) ^2 + \left( \frac{\partial \theta }{\partial Y}\right) ^2}. \end{aligned}$$

In addition, the average Nusselt number ($\overline{Nu}$) can be calculated by integrating the local Nusselt number for vertical left wavy wall which is:

30 $$\begin{aligned} \overline{Nu}_{nf} = \frac{1}{W} \int _{0}^{W} Nu {} \mathrm{{d}}W. \end{aligned}$$

## Numerical method and validation

The Galerkin weighted residual along with finite element methods are employed to investigate the control equations (14)–(18) and Eq. (24) subject to the boundary conditions Eqs. (19)–(22) and Eqs. (25)–(27). The finite element analysis of the momentum equations (15) and (16) is showed using the following procedure:

Initially, we apply the penalty finite element method by excluding the pressure (*P*) including a penalty parameter ($\lambda$) as the following:

$$\begin{aligned} P=-\lambda \left( \frac{\partial U}{\partial X}+\frac{\partial V}{\partial Y}\right) . \end{aligned}$$

This leads to the following momentum equations toward the *X*- and *Y*-directions:

$$\begin{aligned} U\frac{\partial U}{\partial X} + V\frac{\partial U}{\partial Y}{} & {} = \frac{\partial \lambda }{\partial X} \left( \frac{\partial U}{\partial X}+\frac{\partial V}{\partial Y}\right) + \frac{\rho _{f}}{\rho _{nf}} \frac{\mu _{nf}}{\mu _{f}} \frac{1}{Re} \left( {\frac{\partial ^2 U}{\partial X^2} + \frac{\partial ^2 U}{\partial Y^2}}\right) \\{} & {} \quad + \frac{\rho _{f}}{\rho _{nf}} \frac{\sigma _{nf}}{\sigma _{f}} \frac{Ha^2}{Re} \left( V\sin \gamma \cos \gamma - U\sin ^2\gamma \right) , \\ U\frac{\partial V}{\partial X} + V\frac{\partial V}{\partial Y}{} & {} = \frac{\partial \lambda }{\partial Y} \left( \frac{\partial U}{\partial X}+\frac{\partial V}{\partial Y}\right) + \frac{\rho _{f}}{\rho _{nf}} \frac{\mu _{nf}}{\mu _{f}} \frac{1}{Re} \left( {\frac{\partial ^2 V}{\partial X^2} + \frac{\partial ^2 V}{\partial Y^2}}\right) + \frac{(\rho \beta )_{nf}}{\rho _{nf} \beta _{f}} {Ri}\, {\theta } \\{} & {} \quad + \frac{\rho _{f}}{\rho _{nf}} \frac{\sigma _{nf}}{\sigma _{f}} \frac{Ha^2}{Re} \left( U\sin \gamma \cos \gamma -V\cos ^2\gamma \right) . \end{aligned}$$

**Figure 2:**
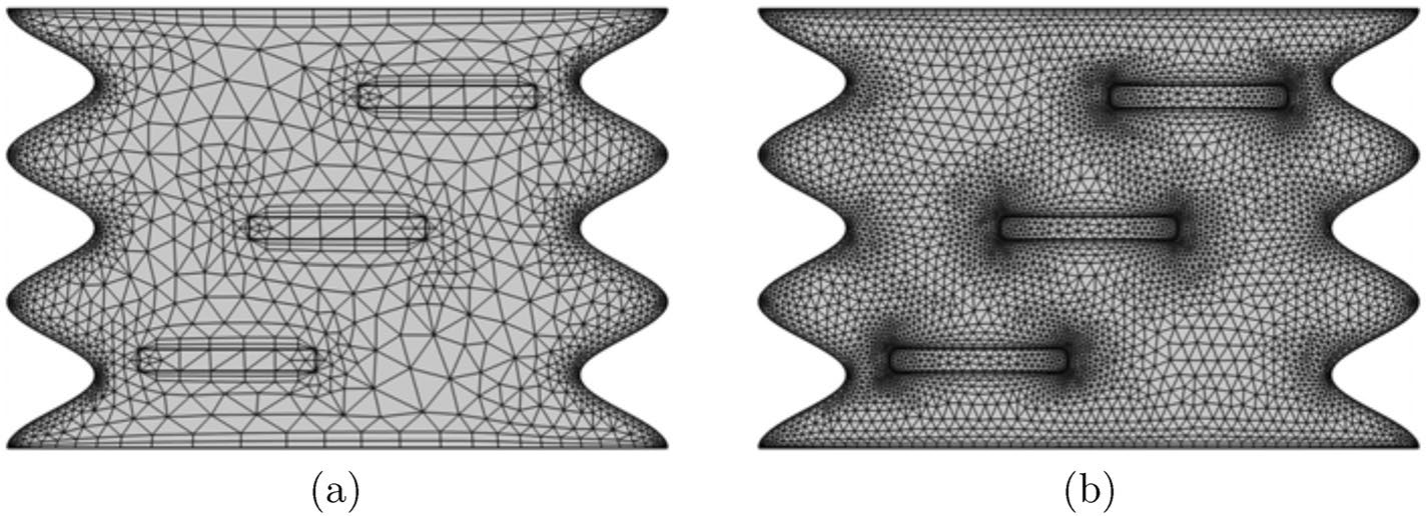
Grid-points distribution for the grid size of (**a**) G1 and (**b**) G2.

The weak (or weighted-integral) formulation with regard to the momentum equations is obtained by multiplying the equation by an internal domain ($\Phi$) and integrating it over the computational domain which is discretised toward small triangular elements as revealed in Fig. 2. The following weak formulations are obtained:

$$\begin{aligned} \int _{\Omega } \left( \Phi _i U^k \frac{\partial U^k}{\partial X} + \Phi _i V^k \frac{\partial U^k}{\partial Y}\right) \mathrm{{d}X\textrm{d}Y}{} & {} = \lambda \int _{\Omega } \frac{\partial \Phi _i}{\partial X} \left( \frac{\partial U^k}{\partial X}+\frac{\partial V^k}{\partial Y}\right) \mathrm{{d}X\textrm{d}Y} \\{} & {} \quad + \frac{\rho _{f}}{\rho _{nf}} \frac{\mu _{nf}}{\mu _{f}} \frac{1}{Re} \int _{\Omega }\Phi _i \left( \frac{\partial ^2 U^k}{\partial X^2} + \frac{\partial ^2 U^k}{\partial Y^2}\right) \mathrm{{d}X\textrm{d}Y} \\{} & {} \quad + \frac{\rho _{f}}{\rho _{nf}} \frac{\sigma _{nf}}{\sigma _{f}} \frac{Ha^2}{Re} \left( \Phi _i V^k \sin \gamma \cos \gamma - \Phi _i U^k \sin ^2\gamma \right) , \\ \int _{\Omega } \left( \Phi _i V^k \frac{\partial V^k}{\partial X} + \Phi _i V^k \frac{\partial V^k}{\partial Y}\right) \mathrm{{d}X\textrm{d}Y}{} & {} = \lambda \int _{\Omega } \frac{\partial \Phi _i}{\partial Y} \left( \frac{\partial U^k}{\partial X}+\frac{\partial V^k}{\partial Y}\right) \mathrm{{d}X\textrm{d}Y} \\{} & {} \quad + \frac{\rho _{f}}{\rho _{nf}} \frac{\mu _{nf}}{\mu _{f}} \frac{1}{Re} \int _{\Omega }\Phi _i \left( \frac{\partial ^2 V^k}{\partial X^2} + \frac{\partial ^2 V^k}{\partial Y^2}\right) \mathrm{{d}X\textrm{d}Y} + \frac{(\rho \beta )_{nf}}{\rho _{nf} \beta _{f}} Ri \int _{\Omega }\Phi _i \theta _{nf}^k\mathrm{{d}X\textrm{d}Y} \\{} & {} \quad + \frac{\rho _{f}}{\rho _{nf}} \frac{\sigma _{nf}}{\sigma _{f}} \frac{Ha^2}{Re} \left( \Phi _i U^k\sin \gamma \cos \gamma -\Phi _i V^k\cos ^2\gamma \right) . \end{aligned}$$

Selection about the interpolation functions as implementing an approximation toward the velocity distribution and temperature distribution as:

$$\begin{aligned}{} & {} U \approx \sum _{j=1}^m U_j \Phi _j(X,Y), \quad V \approx \sum _{j=1}^m V_j \Phi _j(X,Y), \quad \theta \approx \sum _{j=1}^m \theta _j \Phi _j(X,Y). \end{aligned}$$

The nonlinear residual equations for the momentum equations that obtained from the Galerkin weighted residual finite-element method are:

$$\begin{aligned} R(1)_i{} & {} =\sum _{j=1}^{m}U_j \int _{\Omega } \left[ \left( \sum _{j=1}^{m}U_j\Phi _j\right) \frac{\partial \Phi _j}{\partial X} + \left( \sum _{j=1}^{m}V_j\Phi _j\right) \frac{\partial \Phi _j}{\partial Y} \right] \Phi _i\mathrm{{d}X\textrm{d}Y}\\{} & {} \quad + \lambda \left[ \sum _{j=1}^{m} U_j \int _{\Omega }\frac{\partial \Phi _i}{\partial X} \frac{\partial \Phi _j}{\partial X}\mathrm{{d}X\textrm{d}Y} + \sum _{j=1}^{m} V_j \int _{\Omega }\frac{\partial \Phi _i}{\partial X} \frac{\partial \Phi _j}{\partial Y}\mathrm{{d}X\textrm{d}Y}\right] \\{} & {} \quad + \frac{\rho _{f}}{\rho _{nf}} \frac{\mu _{nf}}{\mu _{f}} \frac{1}{Re} \sum _{j=1}^{m}U_j \int _{\Omega } \left[ \frac{\partial \Phi _i}{\partial X}\frac{\partial \Phi _j}{\partial X} + \frac{\partial \Phi _i}{\partial Y}\frac{\partial \Phi _j}{\partial Y}\right] \mathrm{{d}X\textrm{d}Y} \\{} & {} \quad + \frac{\rho _{f}}{\rho _{nf}} \frac{\sigma _{nf}}{\sigma _{f}} \frac{Ha^2}{Re} \left( \sum _{j=1}^{m}V_j \sin \gamma \cos \gamma - \sum _{j=1}^{m}U_j \sin ^2\gamma \right) , \\ R(2)_i{} & {} =\sum _{j=1}^{m}V_j \int _{\Omega } \left[ \left( \sum _{j=1}^{m}U_j\Phi _j\right) \frac{\partial \Phi _j}{\partial X} + \left( \sum _{j=1}^{m}V_j\Phi _j\right) \frac{\partial \Phi _j}{\partial Y} \right] \Phi _i\mathrm{{d}X\textrm{d}Y}\\{} & {} \quad + \lambda \left[ \sum _{j=1}^{m} U_j \int _{\Omega }\frac{\partial \Phi _i}{\partial Y} \frac{\partial \Phi _j}{\partial X}\mathrm{{d}X\textrm{d}Y} + \sum _{j=1}^{m} V_j \int _{\Omega }\frac{\partial \Phi _i}{\partial Y} \frac{\partial \Phi _j}{\partial Y}\mathrm{{d}X\textrm{d}Y}\right] \\{} & {} \quad + \frac{\rho _{f}}{\rho _{nf}} \frac{\mu _{nf}}{\mu _{f}} \frac{1}{Re} \sum _{j=1}^{m}V_j \int _{\Omega } \left[ \frac{\partial \Phi _i}{\partial X}\frac{\partial \Phi _j}{\partial X} + \frac{\partial \Phi _i}{\partial Y}\frac{\partial \Phi _j}{\partial Y}\right] \mathrm{{d}X\textrm{d}Y} \\{} & {} \quad + \frac{(\rho \beta )_{nf}}{\rho _{nf} \beta _{f}} Ri \int _{\Omega } \left( \sum _{j=1}^{m}\theta _j \Phi _j\right) \Phi _i\mathrm{{d}X\textrm{d}Y} \\{} & {} \quad + \frac{\rho _{f}}{\rho _{nf}} \frac{\sigma _{nf}}{\sigma _{f}} \frac{Ha^2}{Re} \left( \sum _{j=1}^{m}U_j\sin \gamma \cos \gamma -\sum _{j=1}^{m}V_j\cos ^2\gamma \right) . \end{aligned}$$

where the superscript *k* is the relative index, subscripts *i*, *j* and *m* are the residual number, node number and iteration number, respectively. For clarifying the nonlinear terms into the momentum equations, a Newton-Raphson iteration algorithm was employed. Convergence of the solution is allowed through relative error should any of the variable satisfies the resulting convergence criteria:

$$\begin{aligned} \left| \frac{\Gamma ^{m+1}-\Gamma ^{m}}{\Gamma ^{m+1}}\right| \le 10^{-5}. \end{aligned}$$

To encourage purpose, we have produced different grid sizes to simulate the minimum flow circulation ($\Psi _{\textrm{min}}$), the average Nusselt number ($\overline{Nu}$) and the processing time (CPU) for the case of $Ha=0$, $Ri=1$, $\phi =0.02$, $N=3$ and $L=1.5$ and the outcome is as exhibited in Table 1. G5 uniform grid is chosen for all computations in this subsection. Average Nusselt number for the current work is validated based on^51^ work for $Ra=10^5$, $A=0.05$, $N=1,\,3$ and $L=1.1$ without magnetic field case as shown in Table 2. As shown in the table, the findings in each scenario are significantly compatible with those in the literature, and these comparisons give credibility to the current approach, which may yield acceptable results.

**Table 1:** Grid sensitivity check at $Ha=0$, $Ri=1$, $\phi =0.02$, $N=3$ and $L=1.5$.

Grid size	Domain elements	$\Psi _{\textrm{min}}$	$\overline{Nu}$	CPU time (s)
G1	7578	− 0.4663	5.9551	12
G2	13506	− 0.4667	6.0347	17
G3	23592	− 0.4660	6.0763	25
G4	55478	− 0.4665	6.2912	55
G5	66846	− 0.4665	6.2902	126

**Table 2:** Comparison of present $\overline{Nu}$ for case $Ha=0$, $\phi =0.0$, $Ra=10^5$ and $L=1.1$.

Amplitude	^51^ at $N=1$	Present at $N=1$	^51^ $N=3$	Present at $N=3$
$A=0.05$	3.68	3.70	3.51	3.55
$A=0.06$	3.57	3.61	3.24	3.30
$A=0.075$	3.43	3.48	2.89	2.99
$A=0.08$	3.38	3.44	2.80	2.91

## Results and discussion

The numerical results of the streamlines, isotherms and heatlines for the following parameter ranges for Richardson number ($0.01 \le Ri \le 10$), Hartmann number ($0 \le Ha \le 50$), nanoparticles volume fraction ($0 \le \phi \le 0.04$), number of undulations ($0 \le N \le 4$) and dimensionless width cavity ($1 \le L \le 2$), along constant thermal conductivity, length and width of the internal solid fins, and Prandtl number of $k_{s}=0.08$, $s=0.05$, $d=0.4$, $\Pr =4.623$, presented. The thermophysical properties of Newtonian fluid (water) and alumina at the reference temperature are listed in Table 3.

**Table 3:** Thermo-physical properties of water with Al$_{2}$O$_{3}$ nanoparticles at $T=310$K^52,53^.

Physical properties	Fluid phase (water)	Al$_{2}$O$_{3}$
$C_p\, \mathrm {(J/kg K)}$	4178	765
$\rho \, \mathrm {(kg/m^3)}$	993	3970
$k\, \mathrm {(W m^{-1} K^{-1})}$	0.628	40
$\beta \times 10^{5}\, \mathrm {(1/K)}$	36.2	0.85
$\mu \times 10^{6}\, \mathrm {(kg/ms)}$	695	–
$d_p\, \text {(nm)}$	0.385	33

**Figure 3:**
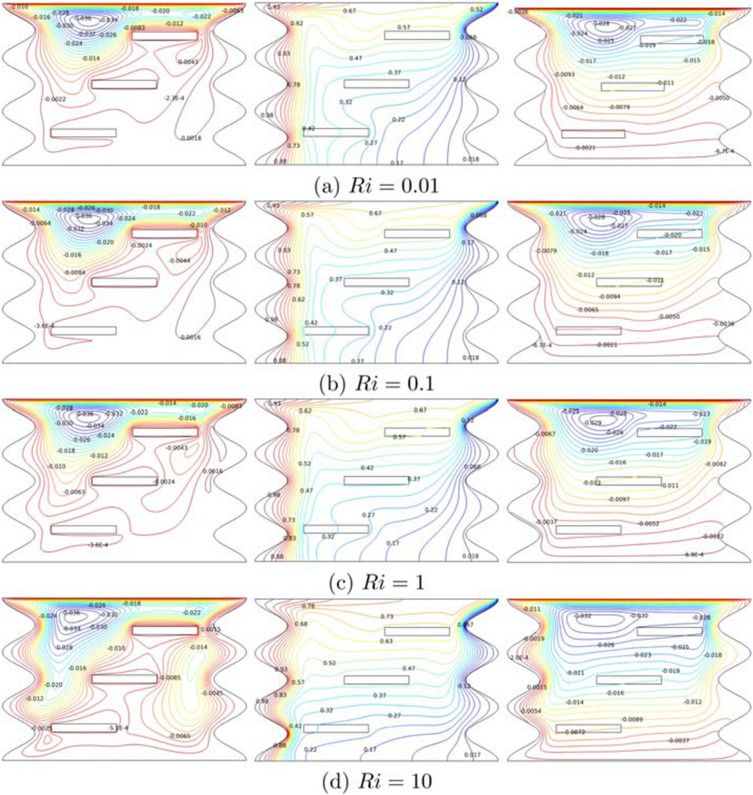
Variations of the (left) streamlines, (middle) isotherms, and (right) heatlines evolution by Richardson number (*Ri*) for $Ha=20$, $\phi =0.02$, $N=3$ and $L=1.5$.

Figure 3a–d illustrates the streamlines, isotherms, and heatlines at Richardson number ($Ri=0.01-10$) for $Re=100$, $Gr=10^5$, $\phi =0.02$, $N=4$, and $L=1.5$. The number of Richardson had influenced in controlling the type of heat convection either natural, forced and mixed. As *Ri* increases, the velocity profile the entire cavity and a small vortex slowly expands clockwise at the upper left cavity. The number of vortices also increased due to raising buoyancy forces from *Ri* and natural convection. As can be seen, the isotherm lines are quite similar from (a) to (d), especially in the cold region concentrating at the top right wavy surface. This happens due to forced convection that pushes the top wall to the right. However, the hot temperatures are seen more concentrated at the wavy bottom part as *Ri* increases. Besides, there is one cell of heatline which starts to enlarge and circulate clockwise in the upper region. Moreover, transportation of heat is fully evolved in the whole cavity, covering both sides’ wave gap by raising *Ri*.

**Figure 4:**
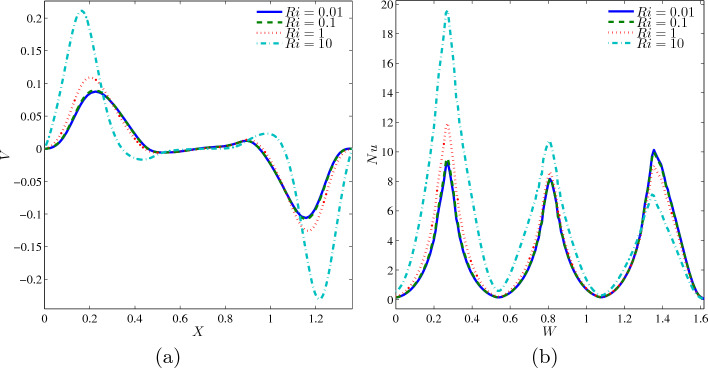
Variations of (**a**) local velocity interfaces with the horizontal line $Y=0.6$ and (**b**) local Nusselt number interfaces with the wavy heater for different *Ri* at $Ha=20$, $\phi =0.02$, $N=3$ and $L=1.5$.

The variations of local velocity interfaces with the horizontal line *Y* at 0.6 for different *Ri* at $Ha=20$, $\phi =0.02$, $N=3$ and $L=1.5$ are shown in the Fig. 4a. The pattern of the graphs in the figure is non-monotonic, in which the fluids had positive velocity at the left part, at rest at the centre, and continue to move in negative velocity at the right part of the cavity. As can be seen, $Ri=10$ precedes along the X-axis due to the buoyancy force that enhances the fluid to move faster. The other values of $Ri=0.01, 0.1$ and 1 are having almost the same pattern of increment and decrement. The reason why $Ri=0.01$ and 0.1 have similar fluctuation is because both scenarios are dominated by the process of forced convection, while $Ri=1$ experienced mid-graph is due to the combined convection. Next, the variation of Nusselt number along the wavy heater for different *Ri* at $Ha=20$, $\phi =$, $N=3$ and $L=1.5$ is demonstrated in Fig. 4b. The presence of buoyancy force influenced $Ri=10$ to host the graph, and followed by $Ri=10$, 1, 0.1 and 0.01 between $W=0--1.3$. However, lower *Ri* of 0.01 and 1 accelerate and lead other graph (Fig. 4b), and this proved that forced convection dominates at the upper part cavity.

**Figure 5:**
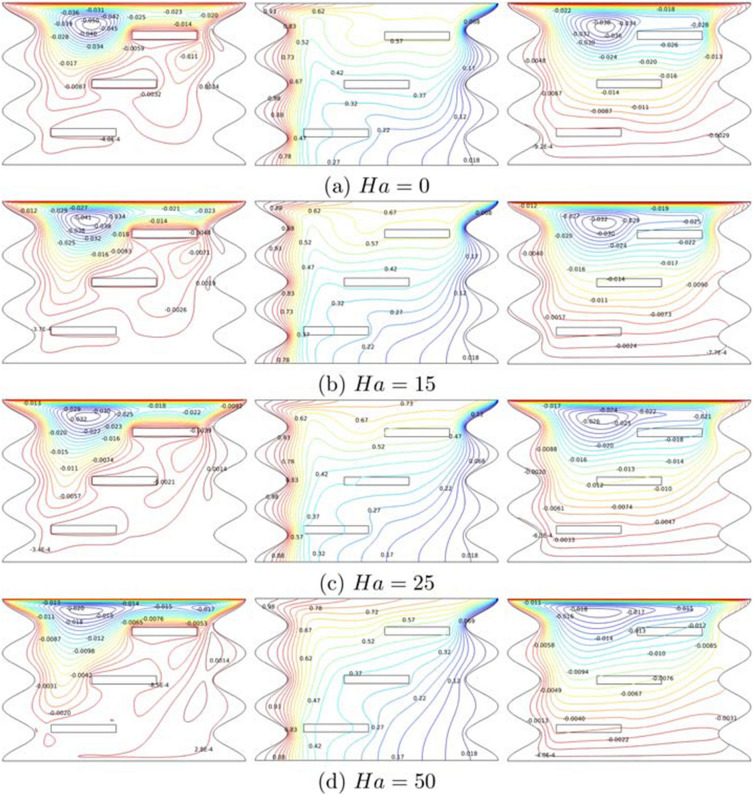
Variations of the (left) streamlines, (middle) isotherms, and (right) heatlines evolution by Hartmann number (*Ha*) for $Ri=1$, $\phi =0.02$, $N=3$ and $L=1.5$.

Figure 5a–d shows the streamlines, isotherms, and heatlines at Hartmann number ($Ha=0-50$) for $Re=100$, $Gr=10^5$, $\phi =0.02$, $N=4$, and $L=1.5$. Hartmann number serves as a magnetohydrodynamic applied in an inclined direction in the cavity. At $Ha=0$, there is no magnetic field implemented, hence the velocity of nanoliquid circulates faster than $Ha=15, 25$ and 50. In addition, the streamlines are accumulated on the upper region of the first fin and gets slower as *Ha* increases. Also, there are plenty of vortices created at $Ha=50$. The top lid-driven triggered forced convection, hence a clockwise primary vortex was produced. Meanwhile, the $45^\circ$ of inclined magnetic field inserted into the cavity does not affect the temperature behaviour much as *Ha* rises. Next, the heat transportation was very similar, along with the increment of *Ha*, except the upper part of the main vortex. A boost in Hartmann number reduces the clockwise rotation but enlarges the size of the vortex. Due to the accelerating impact of the Lorentz force, the center of the eddy lies near the hot wall for magnetic field applied normal to the cold wall. When the buoyancy term and flow shear are both applied at identical strengths, the center of the eddy likewise drifts away from the magnetic flow. The eddies are suppressed by the high magnetic field.

**Figure 6:**
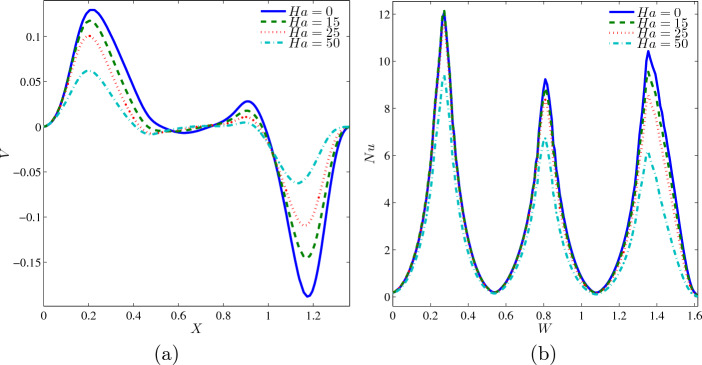
Variations of (**a**) local velocity interfaces with the horizontal line $Y=0.6$ and (**b**) local Nusselt number interfaces with the wavy heater for different *Ha* at $Ri=1$, $\phi =0.02$, $N=3$ and $L=1.5$.

The variation of local interfaces with the horizontal line $Y=0.6$ for different *Ha*, at $Ri=1, \phi =0.02, N=3$ and $L=1.5$ is presented in Fig. 6a. The quadruple graphs are having the same pattern of non-monotonic, which also had forward velocity at the left region that slowly reduces the movement until the remaining heat becomes stationary at some point. However, the graphs slightly increase between $X=0.8$ and 0.9 and drastically plunged backwards velocity. The graph $Ha=0$ succeeded during the analysis by the existence of a magnetic field in the system. Thus, the higher *Ha* weakens the rate of heat transfer, and this is confirmed by $Ha=50$ being the lowest velocity among others. Besides, the variation of local Nusselt number interfaces with the wavy heater for different *Ha* at $Ri=1, \phi =0.02, N=3$ and $L=1.5$ is illustrated in Fig. 6b. The oscillation graphs of four variety of *Ha* obtained three climaxes at $W=0.3$, 0.8 and 1.4 due to the location of the three solid fins in the cavity. Similar to the previous case, $Ha=0$ reaches maximum heat transfer rate because no magnetic field is present and did not interfere with the fluid flow.

**Figure 7:**
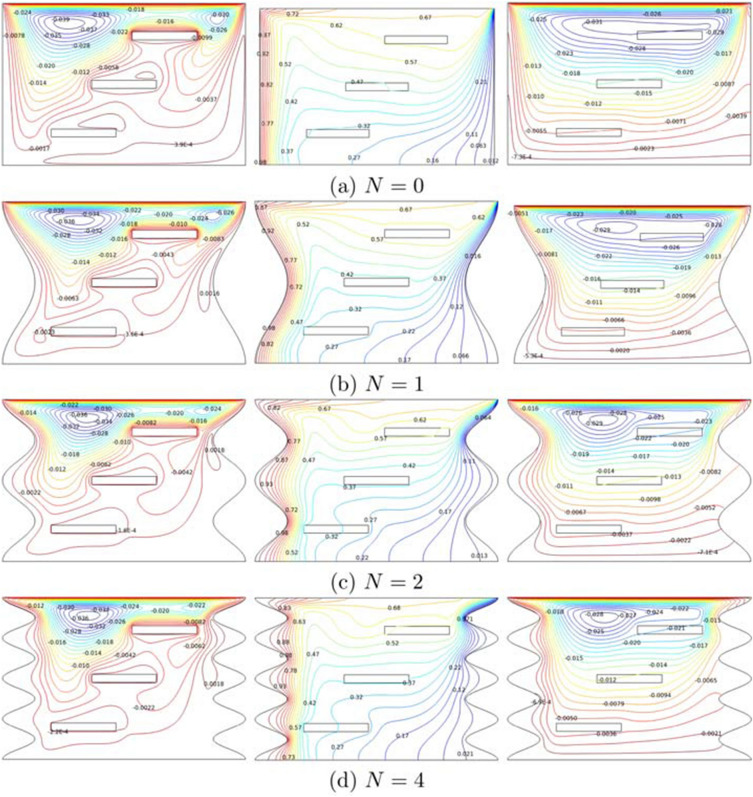
Variations of the (left) streamlines, (middle) isotherms, and (right) heatlines evolution by number of undulation (*N*) for $Ri=1$, $Ha=20$, $\phi =0.02$ and $L=1.5$.

Figure 7 demonstrates the streamlines, isotherms, and heatlines at number of undulation ($N=0-4$) for $Ri=1$, $Ha=20$, $\phi =0.02$ and $L=1.5$. The wavy shape built at both vertical walls developed a wider border and surface of the cavity. The development of undulation compressed the streamline in the cavity, but does not affect the pace of the fluid. There are three main vortices created at the top left and right cavity and circulates near the first fin. As the number of undulation increases, small vortices around the second and third fins diminished since the space cavity began to shrink. Meanwhile, the temperature distribution from the hot to the cold region is neatly arranged with the same symmetry. Four undulations cause the temperature diffusion to strongly squeeze, and the hot temperature is detected at every peak of the wave. Concurrently, there is a gigantic cell of heatline rotating clockwise at the top cavity. The cell slowly deflates, and heat transportation speed gets slower as the number of undulations increased. Because of the large distortion of low temperature at the vertical wavy walls, the heat flow rate near the heater edges decreases as the number of undulations increases. The low temperature from the initial wave crest as it reaches the bottom wall with an increasing amount of undulations.

**Figure 8:**
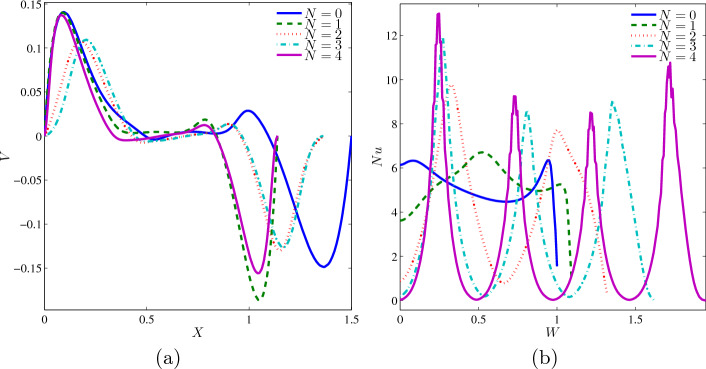
Variations of (**a**) local velocity interfaces with the horizontal line $Y=0.6$ and (**b**) local Nusselt number interfaces with the wavy heater for different *N* at $Ri=1$, $Ha=20$, $\phi =0.02$ and $L=1.5$.

The variation of local velocity interfaces with the horizontal line $Y=0.6$ for different *N* at $Ri=1$, $Ha=20$, $\phi =0.02$ and $L=1.5$ is displayed in Fig. 8a. The fluid’s velocity at $N=0, 1$ and 4 approached $V = 0.15$ at the left region, and the velocity extremely decreased until $X=0.4$ where the fluid is at rest. Then the fluid is revolved backward very quickly at the right region of the cavity. Although these non-monotonic graphs started at the same point, they ended at different spots since $N=0$ provided large space and long width compared to wavy cavity along $Y=0.6$. The variation of local Nusselt number interfaces with the wavy heater for different *N* at $Ri=1$, $Ha=20$, $\phi =0.02$, and $L=1.5$ is as exhibited in Fig. 8b. As more undulations are built at the left wavy heater, the vertical surface becomes wider, and the graph of $N=0$ and 1 reached $W=1$ and 2, respectively. As $N=2, 3$ and 4 generated ascending and descending graphs with the number of peaks depending on the number of undulations. On the other hand, the rectangular cavity ($N=0$) began with a local Nusselt number 6. As it gets higher on the left wall, the heat transfer rate is roughly passive, and gently ascends before $W=1$ but declined sharply at the upper left corner of the cavity. The report shows that heat transfer performance in $N=4$ experienced significant oscillation throughout the wavy heater.

**Figure 9:**
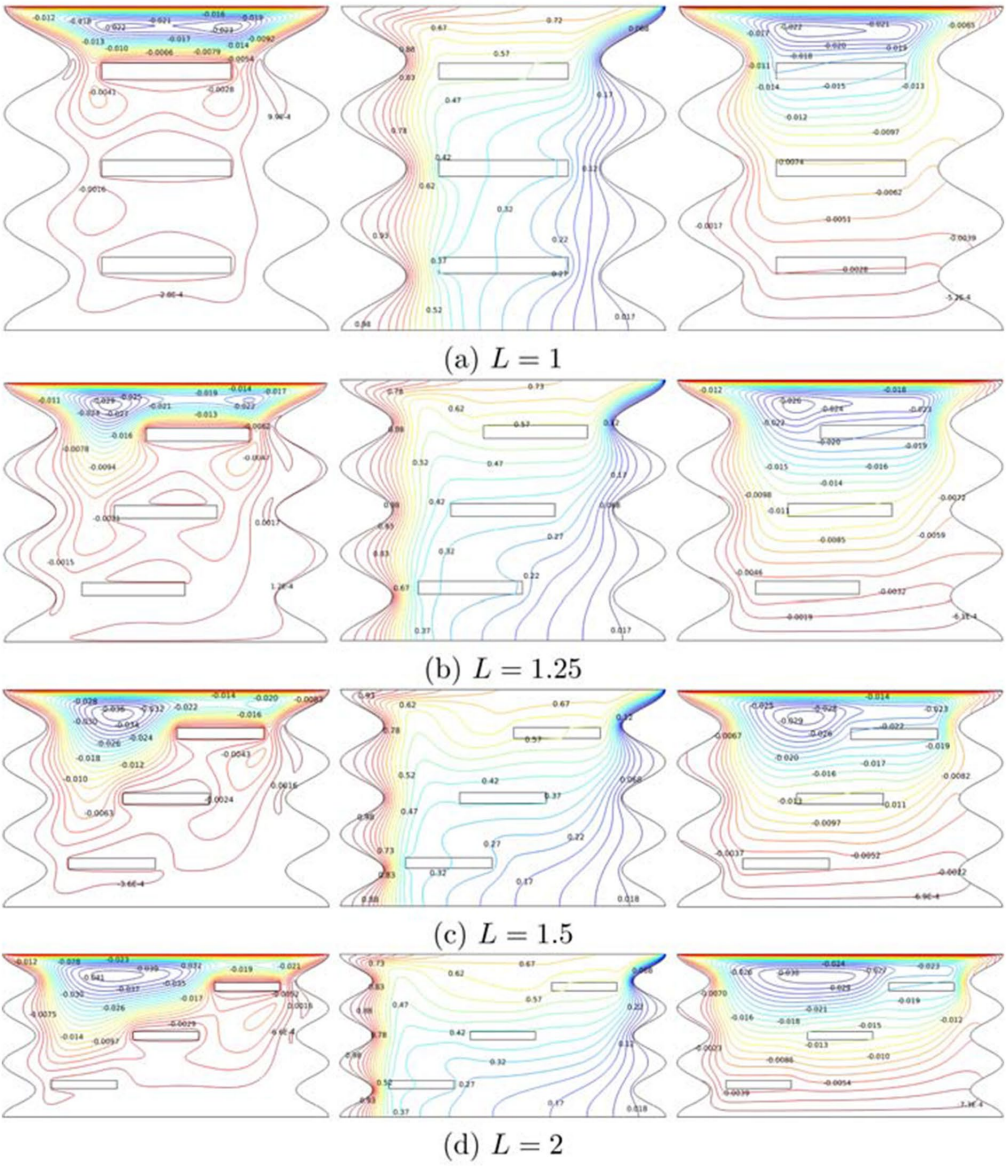
Variations of the (left) streamlines, (middle) isotherms, and (right) heatlines evolution by dimensionless width of the cavity (*L*) for $Ri=1$, $Ha=20$, $\phi =0.02$ and $N=3$.

Figure 9 displays the streamlines, isotherms, and heatlines at dimensionless width cavity ($L=1-2$) for $Ri=1$, $Ha=20$, $\phi =0.02$ and $N=3$. The $L=1$ acts like a square shaped, with three rectangular inner fins placed in tiers in the middle of the cavity. Many eddies generated at $L=1$, of which two identical eddies rotate clockwise at the upper part, three huge eddies circulates the fins, and the last eddy was seen between the second and third fins. Elongation *L* forced the two twin eddies apart; the left vortex enlarging and moved faster while the right vortex began to shrink and revolved slower. The distance from one fin to other fins contributes in modifying the fluids inside the cavity as *L* increases. As can be seen, the growth of *L* does not influence the temperature behaviours that much, but the higher temperature is more attracted to the wave crest. It is obvious from the figure that a primary heatline vortex is generated and started to expand with the augmentation *L*. Also, heat transportation is prompt due to the shorter length and width of the cavity as well as position of the multiple fins.

**Figure 10:**
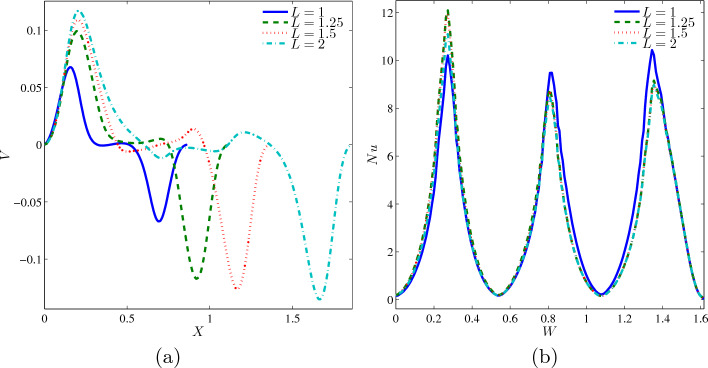
Variations of (**a**) local velocity interfaces with the horizontal line $Y=0.6$ and (**b**) local Nusselt number interfaces with the wavy heater for different *L* at $Ri=1$, $Ha=20$, $\phi =0.02$ and $N=3$.

The variation of local velocity interfaces with the horizontal line $Y=0.6$ for different *L* at $Ri=1$, $Ha=20$, $\phi =0.02$ and $N=3$ is delineated in Fig. 10a. The graphs of $L=1, 1.25, 1.5$, and 2 shows fluctuation patterns with positive velocity, with the fluid pace reduced until stagnant, and after some time, the graph is in the opposite pattern. Small *L* values caused shorter width while the higher *L* values produced long width. The non-monotonic graph for four cases is mastered by $L=2$, followed by $L=1.5, 1.25$, and 1. Besides, the local Nusselt number interfaces with the wavy heater for different *L* at $Ri=1$, $Ha=20$, $\phi =0.02$ and $N=3$ as depicted in Fig. 10b. The oscillating graph of four different L undergoes three peaks at $W=0.3,0.8$ and 1.4. At the bottom left wavy heater, $L=1.25$ and 2 monopolise the first phase, but the graph of $L=1$ took over both of them at the second and third phases of the graph, which are in the middle and top of the vertical wavy heater. The position of inner fins at $L=1$ is closer to the left surface towards the upper cavity compared to other cases, so the high local Nusselt number is conquered.

**Figure 11:**
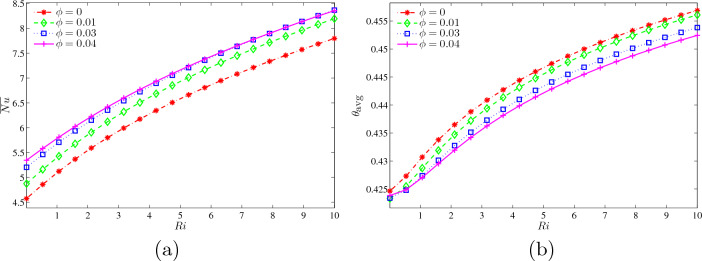
Variations of (**a**) average Nusselt number at the wavy heater and (**b**) dimensionless average temperature with *Ri* for different $\phi$ at $Ha=20$, $N=3$ and $L=1.5$.

Figure 11a shows the average Nusselt number at the wavy heater with *Ri* for different $\phi$ at $Ha=20$, $N=3$ and $L=1.5$. The cavity filled with water without any nanoparticle at $\phi =0$ indicates the slowest rise, in contrast with $\phi =0.01,0.03$ and 0.04. At $Ri=1--4$ which is at forced convection, the local Nusselt number is quite different but it attaches after $Ri=5$ onwards. Next, Fig. 11b illustrates the dimensionless average temperature ($\theta _{\text {avg}}$) with *Ri* for different $\phi$ at $Ha=20, N=3$ and $L=1.5$. The graphs of four different $\phi$ started at low *Ri* which reflect forced convection rose steadily as *Ri* increases until it stops at natural convection of 10. Base fluid without nanoparticle of alumina represented by $\phi =0$ leads the graph because there was a lift of 0.0025 from dimensionless average temperature 0.425 to 0.455. Nonetheless, the highest concentration alumina-nanoparticle, $\phi =0.04$ has the slowest rise as the particles distorts the temperature behaviour in the system.

**Figure 12:**
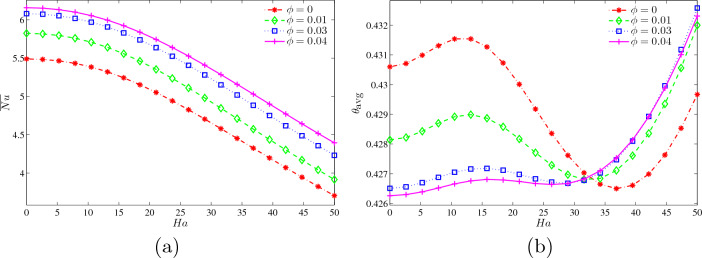
Variations of (**a**) average Nusselt number at the wavy heater and (**b**) dimensionless average temperature with *Ha* for different $\phi$ at $Ri=1$, $N=3$ and $L=1.5$.

Figure 12a presents average Nusselt number at the wavy heater with *Ha* for different $\phi$ at $Ri=1$, $N=3$ and $L=1.5$. Four graphs of $\phi =0, 0.01, 0.03$ and 0.04 declined as number of Hartmann increases. This explains that the increment of Hartmann number will weaken the rate of heat transfer along the wavy surface. Figure 12b demonstrates the dimensionless average temperature with Ha for different $\phi$ at $Ri=1, N=3$ and $L=1.5$ with a monotonic graph. For examples, $\phi =0$ is conducted between $Ha=0$ and 32.5 followed by $\phi =0.01, 0.03$ and 0.04 with different beginnings of $\theta _{\text {avg}}=0.4308, 0.4281, 0.4264, 0.4266$, accordingly. Four of them increased slowly but declined somehow rapidly for $\phi =0$ and 0.01 but declined gradually for $\phi =0.03$ and 0.04. However, all four converged to the same point at $Ha=32.5$ and continued to climb quickly. Now, $\phi =0.04$ and 0.03 monopolised the graph and have the same increment. Graphs are inverted from $Ha=32.5$ to 50, and now the third place is replaced by $\phi =0.01$ and succeeded by $\phi =0$. The reason why $\phi =0.04$ and 0.03 reached the highest dimensionless average temperature is that huge *Ha* produced a great magnetic field and weaken the rate of heat transfer of base fluid but the high concentration of nanofluids in the cavity. When the magnetic field is strong enough, the heated nanofluid particles climb away from the hot wall and partially sweep the wavy wall. Due to the existence of counter rotating eddies, the vortex is moved closer to the wavy walls at high concentration.

**Figure 13:**
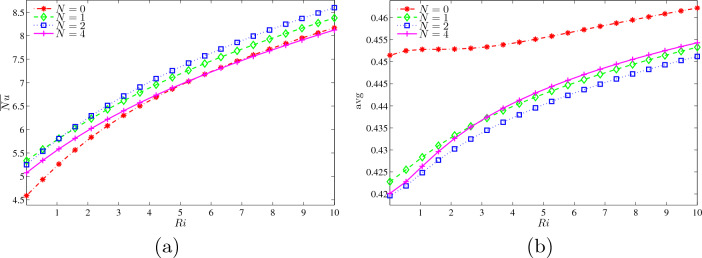
Variations of (**a**) average Nusselt number at the wavy heater and (**b**) dimensionless average temperature with *Ri* for different *N* at $Ha=20$, $\phi =0.02$ and $L=1.5$.

Figure 13a demonstrates the average Nusselt number at the wavy heater with *Ri* for different *N* at $Ha=20$, $\phi =0.02$ and $L=1.5$. As the number of Richardson rises, the heat transfer rate also improves as the number of undulation increases. The average Nusselt number started at 4.6 and 5.1 for $N=0$ and $N=4$, respectively. At $Ri=5$, both graphs reached to $\overline{Nu}=8$ in unison as *Ri* gains. Meanwhile, $N = 1$ and $N=2$ precede $N=2$ initially and grew with the same pace until $Ri=3$. However, they began to disengage, and a graph of $N=2$ led the whole comparison and showed that cavity with two undulations provide better heat performances. Figure 13b demonstrates the dimensionless average temperature with *Ri* for different *N* at $Ha=20, \phi =0.02$ and $L=1.5$. Parameter *N* indicates a wavy surface that has been built at both vertical walls; for that reason, the higher value of *N* narrowed the entire cavity. The graph of $N=0$ lifts slightly from 0.451 until achieving a maximum $\theta _{\text {avg}}=0.46$ along with the augmentation of *Ri*. Besides that, the other undulation numbers of 1, 2 and 4 boosts slowly during forced convection to mixed convection until finishing in natural convection mode.

**Figure 14:**
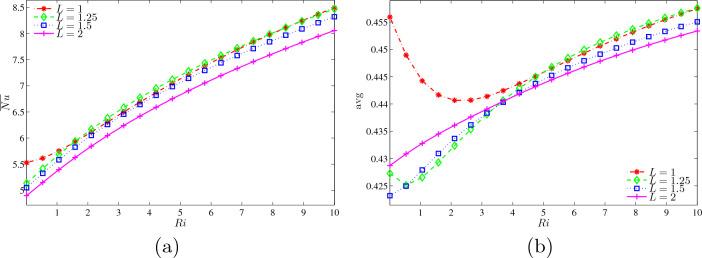
Variations of (**a**) average Nusselt number at the wavy heater and (**b**) dimensionless average temperature with *Ri* for different *L* at $Ha=20$, $\phi =0.02$ and $N=3$.

Figure 14a illustrates the average Nusselt number at the wavy heater with *Ri* for different *L* at $Ha=20$, $\phi =0.02$ and $N=3$. The weakened rate of heat transfer at $L=2$ is due to the lowest rising that started at $\overline{Nu}=4.8$ and ended at 8. Meanwhile, both $L = 1.25$ and 5 have a quite similar increment as number of *Ri* being added. Although $L=1$ monopolised other graphs at lowest *Ri*, it joined the graph of $L=1.25$ onwards until reaching maximum $\overline{Nu}$ of 8.5. Figure 14b illustrates the variations of dimensionless average temperature with *Ri* for different *L* at $Ha=20, \phi =0.02$ and $N=3$. The rise of *L* value, the increased cavity width and the arrangement of the inner fins were gaining further step upwards. The multiple fins are placed vertically at the centre of the cavity, $L=1$, which achieved ultimate of $\theta _{\text {avg}}=0.455$. However, it hits a low of $\theta _{\text {avg}}=0.41$ from $Ri=0$ to 2.5, and then beginning to considerably climb until connecting with the graph of $L=1.25$ until the end of $Ri=10$. Next, $L=1$ declined in the transformation from forced to mixed convection, before rapidly boosts to reach maximum dimensionless average temperature. Other than that, $L=1.5$ and 2 rose moderately starting at $\theta _{\text {avg}}=0.4225$ and 0.429, respectively, with the enlargement of *Ri*.

## Conclusions

The idea of this analysis is to consider inclined magnetohydrodynamics on mixed convection in a rectangular wavy cavity. A movable top lid moves evenly together with different heated wavy surfaces left and right while the other walls were kept adiabatic. There are triple rectangular fins included in the cavity to make this study more interesting and complex. Therefore, the results have been evaluated and observed in terms of streamlines, isotherms, heatlines, local velocity, local and average Nusselt number and dimensionless average temperature. Several conclusions have been made and stated as follows:

1. The addition of nanofluid enhances the heat transfer performance and increases the Richardson number. However, the huge volume fraction of nanoparticle requires more heating time causes delayed increment in dimensionless average temperature.
2. The Hartmann number controls the magnetic field’s strength which badly impacts the heat transfer process. The augmentation of the Richardson number and stronger magnetic field interrupt the nanofluids’ heat transportation. However, a high Hartmann number with high nanofluid’s volume fraction develops dimensionless average temperature in the cavity.
3. The best heat performance for this particular case is to build only two undulations in the model with a significant Richardson number that provided natural convection. Despite this, the ultimate dimensionless average temperature can only be achieved in a basic model of a rectangular cavity, $N=0$.
4. The optimal flow heat transfer rate and the dimensionless average temperature that can obtain the best natural convection in the system is achievable using a square shape of $L=1$.

## Data Availability

All data generated or analyzed during this study are included in this published article.
